# Nicotine Effects and Receptor Expression on Human Spermatozoa: Possible Neuroendocrine Mechanism

**DOI:** 10.3389/fphys.2017.00177

**Published:** 2017-03-28

**Authors:** Rosita A. Condorelli, Sandro La Vignera, Filippo Giacone, Linda Iacoviello, Laura M. Mongioì, Giovanni Li Volti, Ignazio Barbagallo, Roberto Avola, Aldo E. Calogero

**Affiliations:** ^1^Department of Clinical and Experimental Medicine, University of CataniaCatania, Italy; ^2^Department of Biomedical Sciences and Biotechnology, University of CataniaCatania, Italy; ^3^Department of Drug Sciences, University of CataniaCatania, Italy

**Keywords:** nicotinic receptor, spermatozoa, neuroendocrine mechanism, acetylcholine, hexamethonium

## Abstract

The aim of this experimental study was to investigate the mechanism by which nicotine (NIC) alters spermatozoa and to evaluate the expression of nicotinic receptors (nAChR) subunits in human spermatozoa. We analyzed 30 healthy normozoospermic men. Spermatozoa were incubated with NIC 100 ng/ml and the nAChR antagonist, hexamethonium (HEX) (0, 100, 1,000, 10,000 ng/ml) for 3 and 24 h. The following sperm parameters evaluated: (a) progressive motility; (b) mitochondrial membrane potential (MMP); (c) chromatin compactness; (d) externalization of phosphatidylserine (PS); (e) late apoptosis; (f) viability; (g) DNA fragmentation; (h) degree of lipid peroxidation (LP) by flow cytometry; (i) nAChR subunits expression by quantitative Real Time PCR and (j) protein expression evaluation by Western blot analysis. HEX fully antagonized the effects of NIC both after 3 and 24 h of incubation with significant improvement (*p* < 0.05) of sperm progressive motility, MMP, abnormal chromatin compactness, PS externalization, late apoptosis and DNA fragmentation, already at the concentration of HEX 100 ng/ml. The degree of LP increased after incubation with NIC in raw semen but this effect was fully antagonized (*p* < 0.05) by HEX after 3 and 24 h of incubation. Finally, 8 nAChR subunits mRNA (α1, α3, α4, α6, α7, β2, β4, and δ) were found expressed in all samples examined, but only α7 subunit is translated, making an homomer receptor, in non-smokers subjects. The effects of NIC on sperm function are mediated by interaction with a specific nicotinic receptor. The presence of nAChR subunits suggests the presence of a neuroendocrine mechanism on human spermatozoa.

## Introduction

Many evidences attribute to cigarette smoking a negative effect on the reproductive health of both genders, but the mechanisms are not entirely clear. In humans, it has been shown that cigarette smoke is able to alter sperm density, motility, morphology and seminal fluid leukocyte concentration. Effects of the cigarette smoke on sperm DNA integrity, aneuploidy rate, production of free oxygen radicals (ROS) have been evaluated, but the results of these studies appear conflicting: some have shown a negative effect (Stillman et al., 1986; Close et al., 1990; Pacifici et al., 1993; Sofikitis et al., 1995; Vine et al., 1996; Curtis et al., 1997; Rubes et al., 1998; Zhang et al., 2000; Saleh et al., 2002; Kunzle et al., 2003; Said et al., 2005; Sepaniak et al., 2006; Gaur et al., 2007; Reina Bouvet et al., 2007; Calogero et al., 2009; Chohan and Badawy, 2010; El-Melegy and Ali, 2011), while others reported no effect (Vogt et al., 1986; Dikshit et al., 1987; Oldereid et al., 1989; Lewin et al., 1991; Belcheva et al., 2004).

Many compounds derived from cigarette combustion may be responsible for the negative impact that cigarette smoke has on sperm parameters. We have shown that cigarette smoke extract reduces, in a dose- and time-dependent manner, sperm motility and the percentage of spermatozoa with normal mitochondrial function, whereas it increases the percentage of spermatozoa with abnormal chromatin and fragmented DNA (Calogero et al., 2009).

In particular, the effects of nicotine (NIC), an alkaloid present in the tobacco plant and the main constituent of cigarette smoke, on sperm parameters, have been studied, showing that it alters sperm parameters (Pacifici et al., 1995; Reddy et al., 1995; Gandini et al., 1997; Wong et al., 2000; Arabi and Shareghi, 2005).

Interestingly, seminal fluid NIC concentrations are high in men passively exposed to cigarette smoke (10.7 ± 8.5 ng/ml) (Pacifici et al., 1995). Gandini and collaborators, using NIC concentrations similar to those found in the seminal fluid of smokers (70 ng/ml) and 500 times higher (35 μg/ml), after 4 h of incubation, reported a decrease of sperm progressive motility and kinematic parameters (Gandini et al., 1997), even if to a lower extent compared to the decrement found after exposure to cigarette smoke extract (Gandini et al., 1997; Calogero et al., 2009).

We have shown that NIC damages, in a concentration-dependent manner, both conventional and non-conventional sperm parameters (Condorelli et al., 2013). In particular, we evaluated the *in vitro* effects of NIC on sperm motility and non-conventional sperm parameters: mitochondrial function, viability and chromatin/DNA integrity (Condorelli et al., 2013). According to a previous study, we found that NIC decreased progressive motility and the percentage of viable spermatozoa and increased the percentage of spermatozoa with low mitochondrial membrane potential (MMP) and DNA/chromatin integrity in a concentration- and time-dependent manner *in vitro* (Condorelli et al., 2013). In addition, Arabi & Shareghi reported pro-oxidant effects of NIC, with alteration of reduced glutathione cycle and DNA fragmentation which could reverse by antioxidant supplementation (Arabi and Shareghi, 2005).

NIC binds to a class of ionotropic acetylcholine receptors, the “nicotinic receptors” (nAChRs), made up of five subunits. In mammals, 16 different subunits, named α1, α2, α3, α4, α5, α6, α7, α9, α10, β1, β2, β3, β4, γ, δ, and ε, have been identified. The different subunits form a large number of receptor isoforms, allowing a variety of different responses for each tissue. Hexamethonium (HEX) is the main antagonist for the neuronal nAChRs (Mastropaolo et al., 2015), while it does not bind the muscarinic receptors. The neuronal nAChRs bind the acetylcholine, that has been shown to play a role in seminal plasma as described later.

The expression of nAChRs in the posterior post-acrosomal and neck regions of spermatozoa in human sperm have been reported (Kumar and Meizel, 2005). Overall these findings suggest that NIC may interact with its receptor, but only few nAChRs subunits or indirect effects have been investigated and reported. There are no new data since 2005 and some subunits have not yet been researched.

On this basis, the aim of this study was to investigate the mechanism by which NIC exerts its toxic effects on human spermatozoa. To accomplish this, the effects of NIC on sperm motility and non-conventional sperm parameters were evaluated in presence of HEX. Subsequently, the expression of all mammalian nAChR subunits and protein assessment were evaluated in these cells.

## Materials and methods

### Patient selection

The study was conducted on 30 healthy normozoospermic men (mean age 32.2 ± 5.5 years). They did not smoke, they did not drink alcohol and did not use drugs. They had not male accessory glands infection, systemic diseases, microorchidism (testicular volume <12 ml), cryptorchidism, varicocele, and they did not receive hormonal treatment in the last 12 months.

### Sperm preparation

Sperm analysis was conducted according to the WHO 2010 criteria (WHO, 2010). Spermatozoa were separated by swim-up technique. Briefly, an aliquot of seminal plasma was washed with a culture medium with capacitating properties (Biggers-Whitten-Whittingham) supplemented with 20 mM of HEPES and human albumin serum, at 300–500 g for 5–10 min and the supernatant was discarded. Subsequently, 1 ml of BWW was gently added and the samples were left to incubate at 37°C in a CO_2_ incubator for 30–45 min. Spermatozoa with best motility, able to migrate from the pellet, were recovered and used for subsequent experiments.

### Experimental design

On the basis of our previous study (Condorelli et al., 2013) and preliminary data (data not shown) the concentration of 100 ng/ml of NIC was chosen for all experiments. Spermatozoa were incubated with NIC (Sigma-Aldrich S.r.l. Milan, Italy) 100 ng/ml and HEX (Sigma-Aldrich S.r.l. Milan, Italy) (0, 100, 1,000, and 10,000 ng/ml). HEX was added 30 min before the addition of NIC in the culture medium and spermatozoa were incubated for 3 and 24 h at 37°C in an incubator under 5% CO2 atmosphere. The pH values, measured for each experiment, were not significantly different.

At the end of incubation, spermatozoa of the four aliquot were analyzed to evaluate their progressive motility and by flow cytometry, the following parameters: MMP, degree of chromatin compactness, externalization of phosphatidylserine (PS), late apoptosis, viability, DNA fragmentation, and degree of lipid peroxidation (LP). Finally, the expression of nAChR subunits was evaluated by Real-Time PCR (RT-qPCR).

The protocol was approved by the Institutional Review Board, and an informed written consent was obtained from each patient and control.

### Flow cytometric analysis

Flow cytometric analysis was performed using flow cytometer EPICS XL (Coulter Electronics, IL, Milan) equipped with an argon laser at 488 nm. We used the FL1 detectors for the green (525 nm), FL2 for the orange (575 nm) and FL3 for the red (620 nm) fluorescence; 100,000 events (low velocity) were measured for each sample and analyzed by the software Sistem II™, Version 3.0.

#### Evaluation of the MMP

The damage of MMP is an early event of the apoptosis and it is reversible. The MMP reduction can be detected through the use of a lipophilic probe 5,5',6,6'-tetrachloro-1,1',3,3'tetraethyl-benzimidazolylcarbocyanine iodide (JC-1). JC-1 is able to penetrate, selectively, in mitochondria and it exists in monomeric form, emitting at 527 nm; following excitation at 490 nm and in relation to the membrane potential, JC-1 is able to form aggregates emitting at 590 nm. Therefore, the fluorescence changes reversibly from green to orange as soon as the mitochondrial membrane becomes more polarized. In viable cells with normal membrane potential, JC-1 is in the mitochondrial membrane in form of aggregates emitting in an orange fluorescence, while in the cells with membrane potential damaged it remains in the cytoplasm in a monomeric form, giving a green fluorescence.

An aliquot containing 1 × 10^6^/ml of spermatozoa were incubated with JC-1 in the dark, for 10 min, at a temperature of 37°C. At the end of the incubation period, the cells were washed in PBS and analyzed.

#### Assessment of the degree of chromatin compactness

The evaluation of chromatin integrity was performed after the permeabilization of the cell membrane, so as to allow the access of the fluorophore in the nucleus. An aliquot of 1 × 10^6^ spermatozoa was incubated with LPR DNA-Prep Reagent containing 0.1% potassium cyanate, 0.1% NaN_3_, non-ionic detergents, saline and stabilizers (Beckman Coulter, IL, Milan, Italy), in the dark, at room temperature for 10 min and then further incubated with Stain DNA-Prep Reagent containing 50 μg/ml of propidium iodide (PI) (<0.5%), RNase A (4 KUnitz/ml), <0.1% NaN3, saline and stabilizers (Beckman Coulter, IL) in the dark at room temperature. Flow cytometric analysis was performed after 30 min, using FL3 detector.

#### Evaluation of sperm apoptosis/vitality

The exposure of PS on the outer cell surface is an early signal of apoptosis. The assessment of PS externalization was performed using annexin V, an protein that binds selectively to the PS in presence of calcium ions, FITC-labeled. During apoptosis the cells exhibiting the PS even before the loss of semipermeability. Therefore, marking simultaneously the cells with annexin V and PI, we could distinguish: alive (with intact cytoplasmic membrane), apoptotic or necrotic cells. Staining with annexin V and PI was obtained using a commercially available kit (Annexin V-FITC Apoptosis, Sigma Chemical). An aliquot containing 0.5 × 10^6^/ml was suspended in 0.5 ml of buffer containing 10 μl of annexin V-FITC and 20 μl of PI and incubated for 10 min in the dark. After incubation, the sample was analyzed immediately by the detectors FL-1 (FITC) and FL3 (PI). The different pattern of staining allowed to identify the different cell populations: FITC negative and PI negative indicate viable cells, FITC positive and PI negative indicate cells in early apoptosis with cytoplasmic membrane still intact and FITC positive and PI positive indicate cells in late apoptosis.

#### Assessment of DNA fragmentation

The evaluation of DNA fragmentation was performed by the TUNEL method. This uses the TdT (Terminal deoxynucleotidyl Transferase), an enzyme that polymerizes, at the level of DNA breaks, modified nucleotides conjugated to a fluorochrome. The TUNEL assay was performed by using a commercially available kit (Apoptosis Mebstain kit, Beckman Coulter, Milan). To obtain a negative control, TdT was omitted from the reaction mixture; the positive control was obtained pretreating spermatozoa (about 0.5 × 10^6^) with 1 mg/ml of deoxyribonuclease I, not containing RNAse, at 37°C for 60 min prior to staining. The reading was performed by flow cytometry using the FL1 detector.

#### Evaluation of lipoperoxidation

LP evaluation by flow cytometry was performed using the probe, BODIPY (581/591) C11, which after being incorporated into cell membranes, responds to the attack of free oxygen radicals changing its spectrum emission from red to green. This displacement of the emission is shown by the flow cytometer which provides an estimate of the degree of peroxidation. LP was evaluated in two different sperm aliquots of the same patient: the first consisting of spermatozoa separated by swim-up; the second obtained by centrifugation of the seminal fluid (raw semen) About 2 × 10^6^ of spermatozoa were incubated with 5 mM of the probe for 30 min in a final volume of 1 ml. After washing with PBS, flow cytometric analysis was conducted using the FL1 and FL2 detectors.

#### Expression of nAChR subunits

The expression of nAChR subunits was evaluated in 3 samples for each patient; sample A: pellet of raw semen after having discarded the seminal plasma; sample B: pellet after swim-up (total immotile cells) and sample C: motile sperm. Total RNA (1 μg) was amplified by RT-qPCR to identify which nAChR subunit was expressed in the three preparations. Total RNA was isolated using TRIzol (Invitrogen, Carlsbad, CA, USA). First strand cDNA was synthesized using High Capacity cDNA Reverse Transcription Kit (Life Technologies). The RT-qPCR was performed with the SYBR® Green PCR Master Mix (Life Technologies) on a StepOne™ according to the manufacturer's recommended protocol (Life Technologies, USA). Each reaction was run in triplicate. Primers were specifically designed to measure the following nAChR subunits (α1, α2, α3, α4, α5, α6, α7, α9, α10, β1, β2, β3, β4, γ, δ, ε) as previously performed. ^33^The reference gene (housekeeping) was tyrosine 3-monooxygenase/tryptophan 5-monooxygenase activation protein zeta polypeptide (YWHAZ). Subsequently, the samples were analyzed for protein expression evaluation by Western blot analysis. Pellets were resuspended in DPBS and then lysed by sonication. Furthermore, in order to verify the assay, we used as positive control a homogenate of mouse brain. After protein quantification, samples (80 or 100 μg/μl) were mixed with sample loading buffer (Bio-Rad), boiled for 5 min and then loaded into 12% SDSpolyacrylamide (SDS-PAGE) gels and subjected to electrophoresis (120 V, 90 min). The separated proteins were transferred to Immobilon-FL Transfer Membrane (Millipore) using a Trans-Blot Turbo Transfer System (Bio-Rad) (1.2 A, 50 min). After transfer, blots were incubated with Li-Cor Blocking Buffer for 1 h, followed by overnight incubation with 1:1,000 dilution of the primary antibody. Primary polyclonal antibodies directed against AChRa1 (sc-65829), AChRa3 (sc-365479), AChRa4 (sc-74519), AChRa6 (sc-74519), AChRa7 (sc-5544), AChRb2 (sc-11372), AChRd (σχ-390896), and 14-3-3 g (σχ-398423) were purchased from Santa Cruz Biotechnology (Dallas, TX, USA). After washing with DPBS, blots were incubated for 1 h with secondary antibody (1:1,000). Protein detection was carried out using a secondary infrared fluorescent dye conjugated antibody absorbing at 800 nm (catalog number 92632210—Li-Cor Science Tec) or 700 nm (catalog number 92668021—Li-Cor Science Tec). The blots were visualized using an Odyssey Infrared Imaging Scanner (Li-Cor Science Tec).

### Statistical analysis

The results are expressed as mean ± SEM. The data were analyzed by one-way analysis of variance (ANOVA) followed by the Duncan's Multiple Range test. SPSS 22.0 for Windows was used for statistical analysis (SPSS Inc., Chicago, USA). Statistical significance was accepted when the p value was lower than 0.05.

## Results

### Semen parameters

The main sperm parameters of the 30 men enrolled in this study are shown in Table 1. All men had sperm parameters within the normal range according to the WHO 2010 criteria.

**Table 1:** **Sperm parameters (mean±SEM) of the 30 healthy normozoospermic men enrolled in this study**.

**Sperm parameter**	**Values**
Concentration (mil/ml)	90.0 ± 21.0
Total count (mil/ejaculate)	229.5 ± 57.0
Progressive motility (%)	38.0 ± 2.4
Total motility (%)	67.9 ± 1.8
Normal forms (%)	21.1 ± 1.8
Leukocytes (mil/ml)	0.6 ± 0.3

### Effects of nicotine and hexamethonium on sperm progressive motility

**Figure 1:**
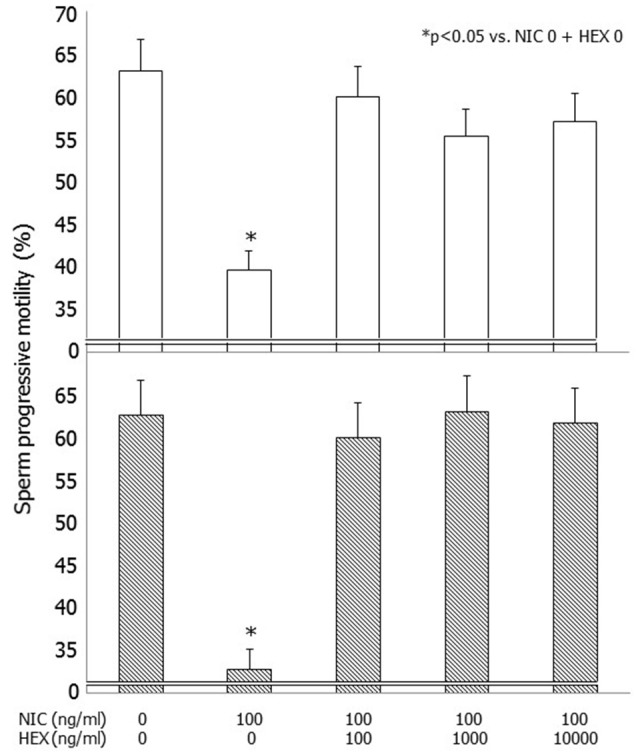
**Effects of graded concentrations of hexamethonium (HEX) (0, 100, 1,000, 10,000 ng/ml) on sperm progressive motility without or with nicotine (NIC) used at the concentrations of 100 ng/ml after 3 (upper panel) and 24 h (lower panel) of incubation**.

NIC (100 ng/dl) decreased sperm progressive motility (*p* < 0.05) after 3 and 24 h of incubation as previously reported (Condorelli et al., 2013) (Figure 1). Pre-incubation with graded concentrations of HEX fully antagonized the effects of NIC both after 3 and 24 h of incubation. This resulted in a significant improvement of sperm progressive motility already at the concentration of HEX 100 ng/ml (*p* < 0.05 vs. NIC 100 ng/ml alone). This antagonistic effect was also observed with the concentrations of 1,000 and 10,000 ng/ml (Figure 1). HEX alone did not have deleterious effects on sperm motility (data not shown).

### Effects of nicotine and hexamethonium on non-conventional sperm parameters

HEX reversed in a statistically significant manner the detrimental effects of NIC, on the percentage of spermatozoa with low MMP, abnormal chromatin compactness, PS externalization, late apoptosis, and DNA fragmentation, already at the concentration of 100 ng/ml (*p* < 0.05 vs. NIC 100 ng/ml alone). These effects were also observed using HEX at the concentrations of 1,000 and 10,000 ng/ml (Table 2). HEX alone had not deleterious effects on all these sperm parameters examined (data not shown).

**Table 2:** **Effects of nicotine and hexamethonium on non-conventional sperm parameters after 3 and 24 h of incubation**.

**Nicotine (ng/ml)**		**0**	**100**	**100**	**100**	**100**
**Hexamethonium (ng/ml)**		**0**	**0**	**100**	**1000**	**10000**
Low mitochondrial membrane potential (%)	3 h	3.7 ± 1.4	16.1 ± 4.7^*^	6.1 ± 2.7	5.2 ± 2.1	6.3 ± 2.4
	24 h	8.1 ± 1.2	32.3 ± 3.1^*^	7.9 ± 1.3	8.3 ± 1.4	9.1 ± 2.1
Abnormal chromatin compaction (%)	3 h	13.1 ± 2.9	19.5 ± 3.6^*^	14.8 ± 3.3	12.8 ± 2.8	14.4 ± 3.2
	24 h	18.1 ± 2.6	28.2 ± 3.1^*^	16.8 ± 2.4	17.8 ± 3.1	17.9 ± 3.3
Alive spermatozoa (%)	3 h	89.8 ± 1.4	81.3 ± 2.3^*^	85.8 ± 2.3	86.6 ± 1.6	86.8 ± 2.2
	24 h	78.6 ± 1.5	65.1 ± 2.1^*^	79.1 ± 3.2	78.1 ± 1.5	76.8 ± 2.1
PS externalization (%)	3 h	1.8 ± 0.5	4.9 ± 1.1^*^	2.8 ± 1.0	2.1 ± 0.6	1.5 ± 0.4
	24 h	3.1 ± 1.2	8.2 ± 1.6^*^	2.7 ± 1.9	2.8 ± 0.8	1.9 ± 0.5
Late apoptosis (%)	3 h	2.7 ± 0.7	4.9 ± 1.1^*^	2.8 ± 1.0	3.2 ± 0.6	3.4 ± 0.9
	24 h	4.3 ± 1.1	8.1 ± 1.3^*^	4.1 ± 1.2	3.9 ± 0.7	4.2 ± 1.3
DNA fragmentation (%)	3 h	2.2 ± 0.4	5.8 ± 0.5^*^	2.1 ± 0.3	2.5 ± 0.9	2.6 ± 0.8
	24 h	3.2 ± 0.3	8.9 ± 1.2^*^	3.1 ± 0.4	3.0 ± 0.8	3.1 ± 0.7

PS: phosphatidylserine,

* *p < 0.05 vs. all groups*.

### Effects of nicotine and hexamethonium on sperm lipoperoxidation

**Figure 2:**
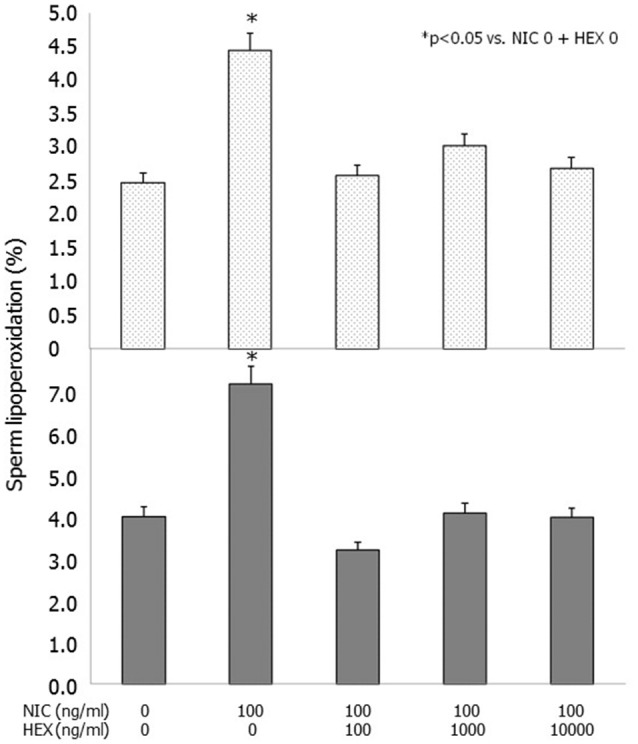
**Effects of nicotine (NIC) (100 ng/ml) and/or graded concentration of hexamethonium (HEX) (0, 100, 1,000, 10,000 ng/ml) on sperm lipoperoxidation after 3 (upper panel) and 24 h (lower panel) of incubation**.

Incubation with NIC (100 ng/ml) resulted in an increased degree of LP compared to control. This difference was statistically significant in raw semen both after 3 and 24 h of incubation (*p* < 0.01 vs. NIC 0), whereas it did not reach the statistical significance when spermatozoa separated by swim-up were incubated (Table 3). HEX fully antagonized the effects of NIC on raw semen after 3 and 24 h of incubation (*p* < 0.05 vs. NIC 100) (Figure 2). HEX alone did not have any statistically significant effect on sperm LP (data not shown).

**Table 3:** **Effects of nicotine on sperm lipoperoxidation after 3 and 24 h in raw semen samples and after swim-up**.

		**Lipoperoxidation (%)**	
		**Nicotine 0**	**Nicotine 100 (ng/ml)**
Raw semen	3 h	0.88 ± 0.25	2.12 ± 0.46^*^
	24 h	1.68 ± 0.49	3.19 ± 0.90^*^
After swim-up	3 h	0.33 ± 0.17	1.33 ± 0.72
	24 h	0.68 ± 0.34	0.98 ± 0.31

* *p < 0.05 vs. Nicotine 0*.

### Semen expression of nAchR subunits

**Figure 3:**
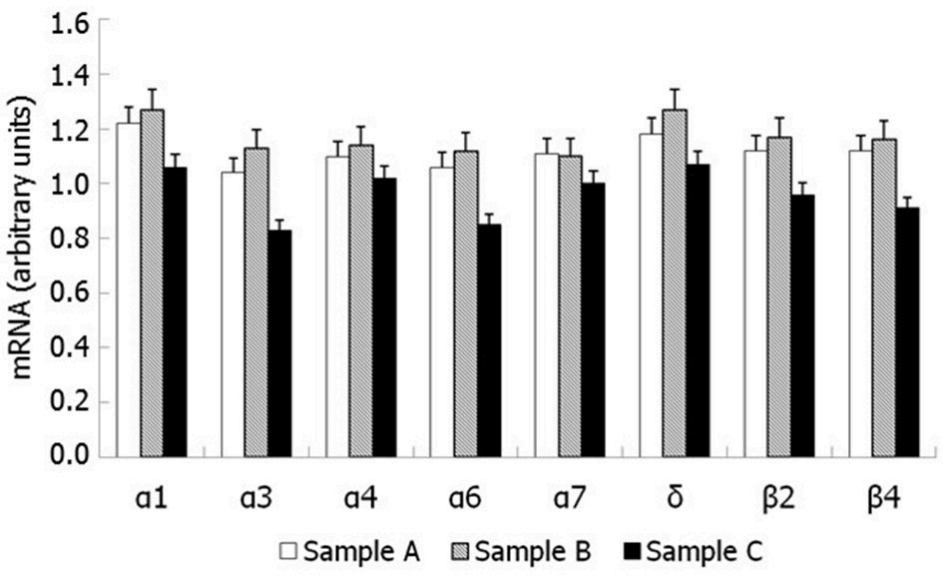
**nAChR subunits mRNA expression by RT-PCR in human spermatozoa**. Sample (A): pellet of raw semen; Sample (B): pellet following swim-up (total immotile cells); Sample (C): motile spermatozoa separated by swim-up.

**Figure 4:**
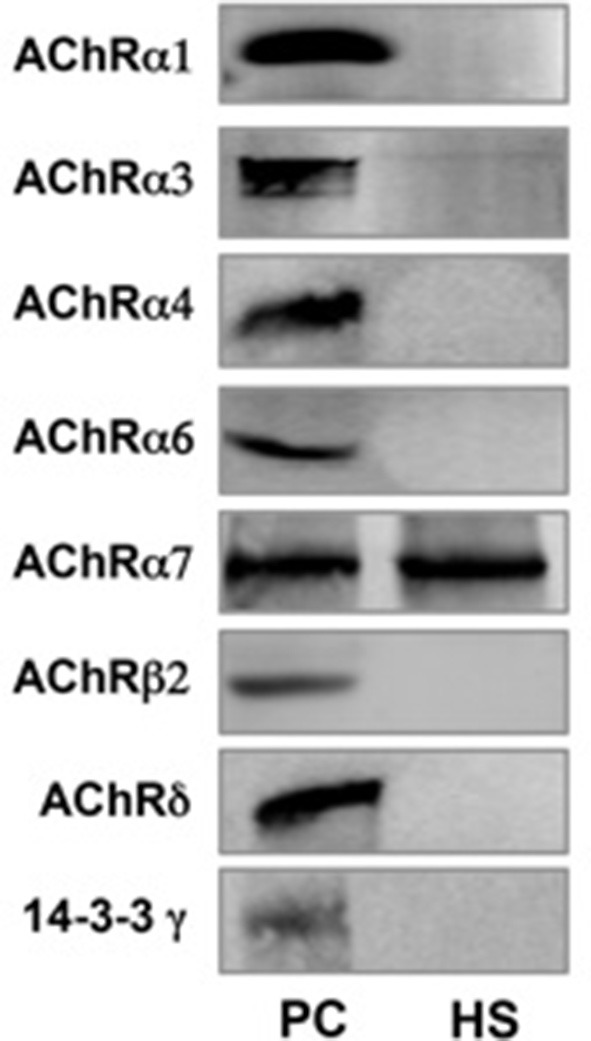
**nAChR subunits expression measured by Western blot in human spermatozoa (PC = Positive control from mouse brain homogenate; HS = Human spermatozoa)**.

RT-PCR showed mRNA expression for 8 nAChR subunits in raw semen (sample A), pellet following swim-up (sample B) and separated motile spermatozoa (sample C). The following subunits were found α1, α3, α4, α6, α7, β2, β4, and δ (Figure 3). Western blot analysis showed that only the nAChR α7 subunit is translated in human capacitated spermatozoa (Figure 4).

## Discussion

The results of the present study confirmed that NIC impairs sperm motility and has a detrimental effect on sperm mitochondrial function, apoptosis and chromatin/DNA integrity. For the first time, we showed that NIC increases significantly the percentage of spermatozoa with LP. This latter effect of NIC reached the statistical significance only in raw semen, but not when a sample of pure spermatozoa was used, suggesting that other cell types, such as leukocytes, though in physiological concentration, contribute to cause a greater LP damage. Indeed, sperm samples selected for this study had a leukocyte concentration within the normal range recommended by the WHO (WHO, 2010 V Edition) manual for sperm analysis (Table 1). All the effects of NIC on sperm function were fully antagonized by co-incubation with the nAChR antagonist HEX, suggesting that they arise from an interaction with its receptor.

The concentration of 100 ng/ml of NIC used for all the experiments of this study was chosen on the basis of previously published concentration-response study (Condorelli et al., 2013) and because it is similar to the NIC concentration found in the seminal fluid of smokers (Pacifici et al., 1993). At this concentration, NIC decreased sperm motility by about 90%. Interestingly, we found that NIC was able to suppress sperm motility already at a concentration about 10 times lower than those present in the semen of men passively exposed to cigarette smoke which has been reported to be about 10 ng/ml (Pacifici et al., 1995; Condorelli et al., 2013). This may explain some cases of “idiopathic” asthenozoospermia in non-smokers.

As a whole, these data suggest that cigarette smoke and, in particular, NIC may cause male infertility by altering sperm motility and some parameters not evident by standard examination of the seminal fluid, such as sperm chromatin/DNA integrity. This parameter seems to play a relevant role in the fertilizing capability of the spermatozoon, the pregnancy rate during assisted reproduction techniques (Sakkas et al., 1996; Host, 2000) and in increasing the incidence of cancers in the offspring (Sorahan et al., 1997). Moreover, the origin of sperm DNA fragmentation is multifactorial: defects in chromatin maturation and oxidative stress lead to a process of apoptosis resulting in an increase of sperm DNA fragmentation (Muratori et al., 2015). So, our data confirm that the NIC increasing oxidative stress can cause not only damage to the sperm chromatin compaction process but also an increase in sperm DNA fragmentation.

HEX was able to counteract the detrimental effects of NIC on all the parameters evaluated in this study. These findings suggest that NIC damages sperm function by interacting with its, not identified in all its isoforms, receptor on the cell surface of human spermatozoa. The nAChR binds with high affinity both nicotine and acetylcholine, its endogenous ligand, but its physiological role is still unclear. Indirect *in-vitro* evidence suggests the presence of the nAChR in human spermatozoa. Indeed, Calzada and colleagues showed that NIC and acetylcholine hyperpolarize sperm plasma membrane by about 20% (Calzada et al., 1992). In addition, this receptor seems to trigger sperm acrosome reaction (AR) since the stimulatory effect of both NIC and acetylcholine is antagonized by pre-incubation with nAChR antagonists (Bray et al., 2002). This evidence is supported by the presence of a α7 receptor in the sperm posterior post-acrosomal and neck region (Kumar and Meizel, 2005) which suggest a role of nAChRs in sperm AR (Bray et al., 2005). The presence of α3, α5, and β4 subunits in the sperm flagellar mid-piece, as α3α5β4 and/or α3β4 receptors, may be important for sperm motility (Kumar and Meizel, 2005).

To evaluate further the presence of all possible nAChRs, we searched for their subunits expression in human spermatozoa. The results showed the mRNA expression of 8 nAChR subunits (α1, α3, α4, α6, α7, β2, β4, and δ) followed by Western blot analysis, thus confirming its presence in these cells. In contrast to what previously reported (Kumar and Meizel, 2005), this study showed the presence of only the α7 nAChR subunit by Western blot analysis.

For this study, non-smokers were selected, since cigarette smoking has been shown to increase the expression of α9 subunit and to decrease that of the δ subunit in the placenta of healthy women smokers. This overexpression is involved in the processes of vasoconstriction, decreased epithelialization and apoptosis observed in placental tissues of smokers (Machaalani et al., 2014). Furthermore, we did not find the expression of α5 and α9 subunits which, on the other hand, was previously reported (Kumar and Meizel, 2005). This may be ascribed to the fact that our patients were non-smokers since cigarette smoking may stimulate the expression of certain subunits and reduce that of others, resulting in different effects on various receptors. In fact, neuronal nAChRs are able to form heteropentameric combinations containing α- and β-subunits as well as homopentameric receptors. The α subunit bind exclusively acetylcholine because it has a specific pocket within which this molecule is placed.

Interestingly, α7 nAChR subunit, as homomer, is widely distributed in the nervous system, especially in neurons of the ciliary ganglia associate with actin (Shoop et al., 2000). Actin is present in the acrosome, post-acrosomal, and neck regions in capacitated human sperm, so this evidence suggest that α7 nAChR subunit may bind the sperm actin cytoskeleton during capacitation (Kumar and Meizel, 2005).

In conclusion, the results of this study suggest that the NIC alters a number of sperm parameters, hence their function, by interacting with a specific nAChR receptor, mainly expressed in the central nervous system. We show that 8 out of 16 nAChR subunits, found to date in mammals, are expressed in human spermatozoa but only α7 subunit is translated, making an homomer receptor, in non-smokers subjects. Therefore, these receptors can be involved with a possible neuroendocrine mechanism not only in sperm AR rate, but also in mitochondrial function, apoptosis, chromatin/DNA integrity, LP and other sperm parameters.

## Author contributions

RC is the principal investigator of this study. AC is the coordinator of all phases of this study. Others authors (SLV, FG, LI, LM, GV, RA, IB) were involved in methodological and statistical aspects.

## Funding

This research did not receive any specific grant from any funding agency in the public, commercial or not-for-profit sector.

### Conflict of interest statement

The reviewer AF declared a past co-authorship with one of the authors AEC to the handling Editor, who ensured that the process met the standards of a fair and objective review. The other authors declare that the research was conducted in the absence of any commercial or financial relationships that could be construed as a potential conflict of interest.
